# The association between perceptions of GenAI-mediated feedback for English pronunciation practice and academic engagement: the mediating role of foreign language enjoyment

**DOI:** 10.3389/fpsyg.2026.1860590

**Published:** 2026-07-09

**Authors:** Si Zhang, Xin Wang, Xiuyun Song, Yanchao Yang, Zhe Peng, Jie Li, Wenjin Yan

**Affiliations:** 1 Faculty of International Languages, Qinggong College, North China University of Science and Technology, Tangshan, Hebei, China; 2 Faculty of Digital and Intelligent Economics, Macau Millennium College, Macao, Macao SAR, China; 3 Institute of International Language Services Studies, Macau Millennium College, Macao, Macao SAR, China; 4 Center for Education and Exchange, Qinggong College, North China University of Science and Technology, Tangshan, Hebei, China; 5 School of English and International Studies, Beijing Language and Culture University, Beijing, China

**Keywords:** academic engagement, English pronunciation practice, foreign language enjoyment, GenAI-mediated feedback, mediation analysis

## Abstract

Drawing on a multi-theoretical framework integrating Self-Determination Theory, Control–Value Theory, and the Broaden-and-Build Theory, this study developed and empirically tested a conceptual model to examine the relationships among perceptions of GenAI-mediated feedback, foreign language enjoyment, and academic engagement. The model specifically investigates the mediating role of foreign language enjoyment. Data were obtained through convenience sampling from 1,701 valid undergraduate participants at a university in northern China. The proposed model was analyzed using the CB-SEM module in SmartPLS 4.0, and mediation effects were assessed via bias-corrected bootstrap confidence intervals based on 5,000 resamples. Results indicated that perceptions of AI-mediated feedback were positively associated with learners’ academic engagement, both directly and indirectly through foreign language enjoyment. These findings highlight the essential role of affective factors in the design and implementation of AI-assisted language learning environments.

## 1 Introduction

The concept of academic emotions was first introduced by the German scholar Pekrun in 2002, referring to the spectrum of emotions directly associated with academic learning, classroom instruction, and academic achievement (Pekrun et al., 2002). Academic emotions encompass a wide range of affective experiences that students encounter throughout the learning process, including enjoyment, boredom, pride, and shame. Among these, enjoyment—a positive, activating, activity-related emotion—has received growing scholarly attention in the field of language acquisition (Chang et al., 2024; Lee, 2020; Li, 2020; Solhi et al., 2024).

According to the Control–Value Theory of achievement emotions, emotions such as enjoyment primarily emerge from learners’ cognitive appraisals of learning activities in terms of perceived control and subjective value (Pekrun, 2006, 2024). Specifically, control appraisal refers to learners’ perceptions of their ability to manage or successfully complete a task, whereas value appraisal pertains to the perceived importance and intrinsic interest of the task. When learners perceive a high level of control over learning activities and consider these activities meaningful and engaging, they are more likely to experience enjoyment (Zhao and Yang, 2023). A substantial body of research has shown that academic enjoyment, as a prototypical positive activating emotion, is strongly associated with various learning-related constructs and is widely recognized as a critical emotional factor in regulating the learning process, including academic performance (Gao, 2025; Grecu et al., 2026; Zeng et al., 2023), learning engagement (Derakhshan and Fathi, 2024; Dewaele and Li, 2021; Li, 2023), and willingness to communicate (Shao, 2025; Zhang and Huang, 2023).

Academic emotions do not arise in isolation; rather, they are collectively shaped by a constellation of proximal factors, including situational perceptions, cognitive appraisals, and external feedback (Forsblom et al., 2022; Khine et al., 2023). Among these, teacher feedback may be understood as an external source of information that is related to students’ sense of control and value through their self-appraisal processes (Khine et al., 2023; Ma et al., 2024). Specifically, feedback regarding student behavior and performance is intrinsically linked to their achievement emotions (Pekrun, 2006, 2017). Furthermore, cumulative feedback on academic failure tends to undermine students’ perceived control, thereby eliciting negative affect, whereas positive feedback can foster positive emotions, which in turn enhance student performance (Khine et al., 2023; Pekrun, 2017; Pekrun et al., 2002). However, prior research has largely focused on interpersonal feedback, such as corrective and encouraging responses provided by teachers or peers (Ma et al., 2024). Notably, such interaction-based feedback may involve a considerable degree of social-evaluative threat. Within the Chinese college English learning context, where the indigenous concept of *mianzi* (social standing) is culturally salient, negative feedback is often perceived as a face-threatening act. Consequently, it can readily evoke evaluation anxiety and trigger defensive, self-protective responses among learners, thereby inhibiting the emergence of enjoyment.

As a fundamental aspect of English phonological development, pronunciation learning encompasses multiple skills, including phoneme discrimination, stress placement, intonation patterns, and connected speech (Yang, 2023). The development of these skills is closely related to precise auditory perception and articulatory imitation, and may also be associated with learners’ affective factors, such as anxiety and concerns about *mianzi*. In the context of college English education in China, pronunciation training is typically conducted through classroom activities such as reading-aloud exercises and oral presentations, which are often accompanied by public teacher evaluation and peer observation. Under the influence of Confucian cultural norms, students tend to be highly sensitive to issues of *mianzi* and evaluative pressure. When pronunciation errors are corrected publicly, learners are prone to heightened nervousness and may adopt avoidance behaviors, a phenomenon commonly referred to as “mute English” (He, 2013). In essence, learners choose oral silence over the risk of making mistakes, sacrificing communicative practice to safeguard their *mianzi*. Such a high-threat learning environment may not only limit opportunities for practice and pronunciation development but also reduce learners’ positive emotional experiences, particularly enjoyment, during classroom participation. As a result, learners’ willingness to engage and their active involvement in pronunciation-related tasks may be undermined. In other words, although traditional classroom pronunciation feedback can support learning improvement, the psychological pressure associated with public evaluation may simultaneously inhibit the emergence of enjoyment, making it difficult for learners to sustain active engagement in pronunciation practice.

Against this backdrop, the advent of generative artificial intelligence (GenAI) technologies has introduced GenAI-mediated feedback as a novel form of external environmental stimulus. Serving as either an alternative or a complement to interpersonal feedback, it provides learners with a distinct pathway from traditional human interaction, particularly in EFL pronunciation and speaking practice, where learners often require repeated feedback and low-anxiety learning environments (Mompean, 2024; Shafiee Rad and Roohani, 2024; Zhong et al., 2025).

Compared with public feedback in classroom settings for English pronunciation studies, AI-generated feedback offers notable advantages in both precision and personalization (Zhong et al., 2025). These features allow learners to efficiently identify their individual pronunciation difficulties and engage in targeted practice, thereby facilitating observable improvement and enhancing their sense of competence. At the same time, learners can independently control the content, timing, frequency, and pace of practice, further strengthening their sense of autonomy. Moreover, the parasocial and private nature of AI-mediated interaction creates a low-threat learning environment that simulates interpersonal communication while minimizing social pressure (Zhong et al., 2025). Within such environments, learners experience a reduction in fear of negative evaluation. Moreover, sustained engagement with personalized feedback fosters relatedness and psychological safety, effectively neutralizing threats to their *mianzi* and alleviating the cognitive demands of self-protection (Ding and Muhyiddin B Yusof, 2025; Lee and Hsieh, 2019).

According to the Control–Value Theory (Pekrun, 2006, 2024), improvements in learners’ sense of competence, together with their autonomy in controlling the pace of learning and selecting practice content, jointly enhance learners’ control appraisal of pronunciation learning tasks. Simultaneously, the personalized nature of AI feedback, the visibility of immediate progress, and its precise alignment with individual pronunciation difficulties further strengthen learners’ value appraisal of the task, reinforcing both its perceived importance and intrinsic interest. Moreover, through parasocial interaction and private feedback mechanisms, AI-mediated feedback transforms the pronunciation learning environment from a “high-threat” to a “high-relatedness” context, effectively reducing the cognitive demands associated with *mianzi*-related concerns. The establishment of such psychological safety enables learners to redirect cognitive resources away from anxiety-driven self-protective behaviors toward greater self-regulation and task control. By enhancing control appraisal, learners are encouraged to engage in deeper language experimentation and more active attempts at pronunciation production. Taken together, from the perspective of the Control–Value Theory, these conditions collectively reinforce learners’ control and value appraisals of pronunciation learning tasks, which serve as proximal antecedents of achievement emotions.

Hence, pronunciation learning places heightened demands on immediate correction, low-threat learning environments, and personalized guidance. This underscores the considerable potential of generative AI–mediated pronunciation feedback to enhance learners’ enjoyment, psychological safety, and willingness to engage in practice. Accordingly, systematic research is needed to evaluate the effectiveness of AI-based pronunciation feedback in university EFL pronunciation learning.

Therefore, the present study proposes a model examining the relationship between GenAI-mediated pronunciation feedback and learning engagement, with a focus on the mediating role of academic enjoyment. Grounded in the Control–Value Theory, the model conceptualizes learners’ perceptions of GenAI-mediated pronunciation feedback as the independent variable, academic enjoyment as the mediating variable, and learning engagement as the outcome variable.

This focus is also consistent with the affective turn in applied linguistics, which has emphasized that emotions are central to understanding language learning and teaching rather than merely peripheral affective factors (Richards, 2022). In particular, positive emotions may support learners’ willingness to participate, experiment, communicate, and engage in autonomous learning. The present study also responds to Zarrinabadi’s (2025) call for futures-oriented inquiry in language education by examining how GenAI-mediated feedback is associated with learners’ emotional experiences and academic engagement in EFL learning.

The present study contributes to the growing literature on AI-assisted language learning by extending research beyond the cognitive and performance-related outcomes of use to learners’ emotional experiences. Specifically, by examining the mediating role of foreign language enjoyment, the study provides further evidence for the importance of positive emotions in understanding how students’ perceptions of GenAI-mediated feedback are associated with academic engagement. In doing so, the study enriches current understanding of the interplay among technological feedback, affective factors, and learner engagement in foreign language learning contexts.

## 2 Literature review

### 2.1 Feedback

According to Hattie and Timperley (2007), feedback is conceptualized as information delivered by an agent concerning specific facets of an individual’s performance or comprehension. Feedback exerts a profound influence on academic growth and achievement. Feedback is classified into various typologies based on distinct factors. Early models, such as Brookhart’s (2008), categorized feedback by focusing on the dimensions of timing, amount, mode, and audience. Subsequent research expanded this scope by investigating the source and function of feedback as pivotal variables (Çevikbaş and Argün, 2016). More recently, a new research paradigm has emerged that integrates both frameworks to analyze feedback through a more comprehensive set of variables (Tasdemir and Yalcin Arslan, 2018), specifically examining: (a) necessity, (b) frequency, (c) timing, (d) type of error to be corrected, (e) types of feedback, and (f) source.

Traditionally, feedback research has primarily focused on human-mediated sources, particularly teacher feedback (Gentrup et al., 2020; van der Lans et al., 2018), peer feedback (Huisman et al., 2018), and parental feedback (Segal et al., 2021). Numerous studies have demonstrated that effective human feedback can enhance learners’ academic performance, motivation, self-efficacy, and engagement by providing guidance, encouragement, and opportunities for self-regulation. With the rapid advancement of artificial intelligence technologies, however, researchers have increasingly begun to explore AI-mediated feedback as a novel and potentially transformative source of educational support (Zhong et al., 2025). Compared with traditional human feedback, AI-generated feedback offers several distinctive advantages, including immediacy, scalability, personalization, and continuous availability. Compared with traditional human feedback, AI-generated feedback offers several distinctive advantages, including immediacy, scalability, personalization, continuous availability, and enhanced privacy. In particular, AI-mediated feedback allows learners to practice and receive corrective information in a relatively private and low-pressure environment, which may reduce embarrassment, face concerns, and fear of negative evaluation commonly associated with human-mediated feedback.

### 2.2 Foreign language enjoyment

Over the past few decades, affective research has experienced a significant paradigm shift. Traditionally, the field was dominated by a “negativity bias,” with primary focus on Foreign Language Anxiety as a key barrier to learning (Dewaele and Macintyre, 2014; Horwitz et al., 2010). However, with the emergence of Positive Psychology in applied linguistics, scholars have increasingly shifted their focus toward learners’ positive emotional experiences.

Among these, enjoyment has emerged as a cornerstone construct. Researchers (Dewaele and Macintyre, 2014) have introduced Foreign Language Enjoyment as a multidimensional positive affective state. It also refers to pleasant emotions that arise when individuals move beyond their usual equilibrium, stretch their abilities, and engage in new experiences, particularly while dealing with challenging tasks (Elahi Shirvan et al., 2020). From a related perspective, Botes et al. (2021) defined Foreign Language Enjoyment (FLE) as a context-specific positive affect that emerges from successfully navigating complex language challenges, serving to fulfill students’ psychological needs within the instructional setting. Meanwhile, Boudreau et al. (2018) distinguished enjoyment from mere pleasure, arguing that FLE goes beyond superficial gratification and instead manifests through deep intellectual engagement, heightened attention, and the pursuit of optimal challenges. In essence, although these definitions differ in emphasis, they collectively portray foreign language enjoyment as a high-arousal, growth-oriented emotion that unites cognitive challenge with psychological satisfaction.

### 2.3 Academic engagement

Academic engagement represents the quality of student effort and involvement committed to activities with clear academic and educational objectives (Prakat et al., 2020). The dominant literature typically adopts the three-dimensional framework (Fredricks et al., 2005), which conceptualizes academic engagement as a multidimensional construct consisting of behavioral, emotional, and cognitive engagement. Behavioral engagement refers to students’ observable participation in academic activities, including attendance, classroom interaction, task completion, and adherence to classroom rules. Emotional engagement captures students’ affective responses toward school, teachers, peers, and learning tasks, such as feelings of interest, enjoyment, belonging, or anxiety. Cognitive engagement represents the deepest level of involvement and reflects the extent to which students are willing to invest psychological effort in mastering complex knowledge and skills. It is typically manifested through the use of metacognitive strategies, self-regulated learning, and a willingness to exceed minimal academic requirements. Subsequent research has further expanded this framework by introducing agentic engagement (Reeve and Tseng, 2011), which highlights students’ proactive contributions to the learning process. From this perspective, students can be understood as active participants in the learning process, as they may express preferences, ask questions, or request clarification during classroom activities. The inclusion of agentic engagement thus offers a more comprehensive understanding of how students actively shape their learning environments.

### 2.4 The relationship between foreign language enjoyment and academic engagement

From a theoretical perspective, the relationship between foreign language enjoyment and academic engagement can be explained by the Broaden-and-Build Theory of Positive Emotions (Fredrickson, 2001, 2004). This theory posits that positive emotions influence individuals’ cognition and behavior through a “broaden-and-build” mechanism. Specifically, positive emotions initially broaden individuals’ momentary thought–action repertoires, enhancing openness and flexibility in cognitive processing, problem-solving, and behavioral choices. In learning contexts, when learners experience positive emotions such as enjoyment, they are more likely to develop interest in learning activities and demonstrate stronger exploratory tendencies and interactional motivation, thereby engaging more actively in classroom activities. Furthermore, the “build” component of the theory suggests that repeated experiences of positive emotions gradually accumulate into enduring personal resources, such as cognitive flexibility, self-confidence, and positive social relationships. These resources, in turn, further support learners’ sustained participation and engagement in classroom learning.

A substantial body of studies has demonstrated a significant positive relationship between foreign language enjoyment and learners’ academic engagement (Derakhshan and Fathi, 2024; Jin, 2024; Lin and Wang, 2025; Wang, 2022), indicating that learners who experience higher levels of enjoyment in foreign language classrooms tend to display greater classroom engagement, such as participating more actively in classroom interactions, maintaining stronger focus on learning tasks, and investing greater effort in language learning activities. For instance, Jin’s study (Jin, 2024), drawing on Self-Determination Theory, confirmed that foreign language enjoyment may serve as an important emotional resource that is positively associated with classroom engagement among Chinese EFL students, potentially showing a stronger association than L2 grit. Therefore, this study proposes Hypothesis 1 (H1):

*H1*: Foreign language enjoyment is positively correlated with students’ academic engagement.

### 2.5 The relationship between feedback and academic engagement

The provision of educational feedback has long been regarded as a highly effective instructional approach for improving student learning outcomes (Qi et al., 2024). Feedback is understood as information delivered to learners regarding their performance, intended to guide and shape their subsequent study behaviors (Ambrose, 2010). Feedback can be delivered verbally or in written form (Lipnevich and Panadero, 2021) and may originate from various sources, including peers (Cao et al., 2022), parents (Segal et al., 2021), teachers (Dong et al., 2021; He and Wen, 2025; Pan and Shao, 2020), or technological tools (Okumuş Dağdeler, 2025; Zhong et al., 2025). In EFL contexts, feedback is often tailored to specific language skills, particularly writing (Daşer, 2024) and speaking (Zhong et al., 2025). Furthermore, a growing body of empirical evidence has demonstrated a robust correlation between the provision of feedback and student engagement (Dong et al., 2021; He and Wen, 2025; Pan and Shao, 2020). Research indicates that high-quality feedback does not merely inform students of their progress but actively fosters their engagement in the learning process.

Therefore, this study proposes Hypothesis 2 (H2):

*H2*: GenAI-mediated feedback for English pronunciation practice is positively correlated with students’ academic engagement.

### 2.6 The relationship between feedback and foreign language enjoyment

The positive relationship between feedback and foreign language enjoyment can be profoundly elucidated through the integrated perspective of Self-Determination Theory (Deci and Ryan, 1985) and Control–Value Theory (Pekrun, 2006, 2024). First, from the perspective of Self-Determination Theory, AI-mediated feedback satisfies learners’ three basic psychological needs. The precision and personalization of AI feedback help learners overcome specific pronunciation difficulties, directly enhancing their sense of competence. By allowing learners to independently determine the content, timing, and pace of practice, AI promotes a high degree of autonomy. Furthermore, the private and parasocial nature of AI interaction creates a low-threat environment that fosters a sense of relatedness and psychological safety, effectively mitigating social pressure and *mianzi* concerns. Subsequently, according to Control–Value Theory, the fulfillment of these psychological needs translates into positive cognitive appraisals of the learning task. Improvements in competence and autonomy strengthen learners’ control appraisal—the perception of being in command of their learning outcomes. Simultaneously, the visibility of immediate progress and the precise alignment with individual needs enhance their value appraisal—the perception of the task’s importance and intrinsic interest. As these two appraisals constitute the proximal antecedents of achievement emotions, their combined strengthening redirects cognitive resources from anxiety-driven self-protection toward active self-regulation, thereby significantly fostering and sustaining foreign language enjoyment.

A substantial body of empirical research consistently demonstrates a robust positive correlation between traditional human feedback and foreign language enjoyment (Ma et al., 2024; Yunita et al., 2022). Recently, exploratory research within AI-supported learning environments has also emerged, providing preliminary evidence that AI-generated feedback can positively influence foreign language enjoyment (Zhang et al., 2024; Zhao, 2025). For instance, Zhang et al. (2024) conducted a 6-week quasi-experimental study involving 131 Chinese English majors randomly assigned to an experimental group (*n* = 65) and a control group (*n* = 66). By utilizing pre- and post-test questionnaires to evaluate the impact of AI voice assistants, C. Zhang found that the experimental group experienced a significant increase in foreign language enjoyment and willingness to communicate, alongside a marked decrease in language anxiety. These findings confirm the positive role of AI tools in fostering positive emotions and reducing psychological barriers in language learning.

Therefore, this study proposes Hypothesis 3 (H3):

*H3*: GenAI-mediated feedback for English pronunciation practice is positively correlated with students’ foreign language enjoyment.

**FIGURE 1:**
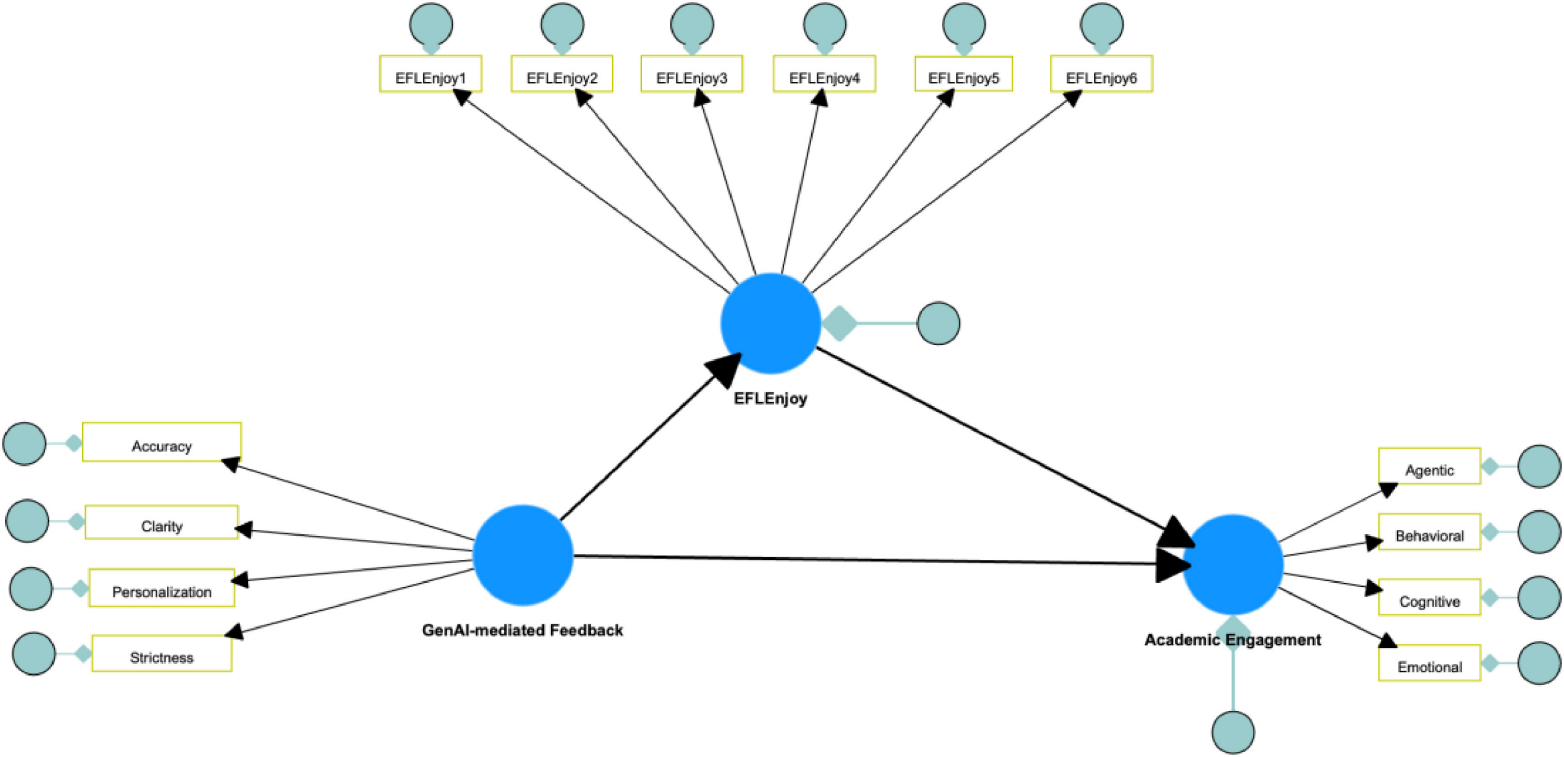
Conceptual model.

Based on the theoretical framework and empirical evidence outlined above, the present study proposes a mediation model as shown in Figure 1 to examine the mechanisms underlying the relationship between GenAI-mediated feedback and students’ academic engagement. In this model, learners’ perceptions of GenAI-mediated feedback serve as the independent variable, student engagement as the outcome variable, and foreign language enjoyment as the mediating variable.

## 3 Methods

### 3.1 Ethical considerations

This study received ethical approval from the Institutional Review Board of Qinggong College, North China University of Science and Technology (IRB No. QGXYLL20240003) and was conducted in strict accordance with the Declaration of Helsinki. Using a convenience sampling method via WeChat and QQ workgroups, teachers initially distributed the survey link, which was subsequently forwarded to students by their peers. Data collection was conducted on the Wenjuanxing platform from December 3 to December 27, 2025, with each session lasting approximately 3–5 min. To ensure informed consent, the survey explicitly detailed its purpose, the voluntary nature of participation, and confidentiality measures. Participants were assured that participation would not affect their course grades. An initial filter question required participants to confirm their informed consent and voluntary participation before proceeding with the survey. The present study represents one component of a larger longitudinal research project. Although student ID numbers were collected for the purpose of matching participants across multiple waves of data collection and for the purpose of matching academic records, they were not used in any analyses reported in the present study.

### 3.2 Participants

Participants were recruited using a convenience sampling strategy from the first author’s institution. All participants were first-year undergraduate students enrolled in compulsory College English courses at the time of data collection. The coursebook used in their English classes was 21st Century College English (Book 1), published by Fudan University Press. As no standardized English proficiency test was administered as part of the study, participants’ English proficiency levels could not be objectively determined. However, based on their educational background and placement in the university English curriculum, they were generally considered to possess an intermediate level of English proficiency, approximately approaching the level expected prior to taking the College English Test Band 4 (CET-4) in China. Information regarding participants’ prior language-learning experiences was not collected.

Of the 1,930 students who accessed the questionnaire, 1,755 submitted initial responses. During data screening, participants’ self-reported dates of birth were examined to identify implausible age information. Some responses indicated birth years between 2016 and 2025, which would imply an age below 10 years and was therefore clearly inconsistent with the demographic characteristics of university students. These cases were reviewed and judged to reflect careless or invalid responding rather than genuine demographic information. Accordingly, 54 responses with implausible ages below 10 years were excluded from the final analysis. No separate attention-check items were included; instead, the implausible age criterion was used as an objective data-quality indicator because it was based on self-reported demographic information that clearly contradicted the eligibility characteristics of the target sample. After excluding these invalid responses, the final sample comprised 1,701 participants. The demographic breakdown was as follows: 794 males (46.7%) and 907 females (53.3%); 422 participants (24.8%) were of urban origin, while 1,279 (75.2%) were of rural origin.

### 3.3 Instruments

#### 3.3.1 Scale for students’ attitudes toward AIGC feedback in English pronunciation learning

AI-mediated English pronunciation was assessed using the Scale for Students’ Attitudes Toward AIGC Feedback in English Pronunciation Learning (Zhong et al., 2025). The scale measures four key constructs through 16 items: Accuracy, Strictness, Clarity, and Personalization. Each dimension, comprising four items, evaluates students’ perceptions of the system’s diagnostic precision, feedback rigor, comprehensibility, and adaptive tailoring, respectively. All items employed a 5-point Likert scale (1 = strongly disagree; 5 = strongly agree). For the structural model, the average score of each dimension was used as an observed indicator for the corresponding first-order construct. The instrument demonstrated high reliability in the present study. In the present study, the CFA results indicated good model fit: χ^2^(98) = 724.930, *p* <0.001, χ^2^/df = 7.397, RMSEA = 0.061, SRMR = 0.008, GFI = 0.950, AGFI = 0.930, NFI = 0.986, TLI = 0.985, and CFI = 0.988. Overall, these results support the adequacy of the proposed measurement model. The constructs demonstrated excellent reliability and convergent validity. Cronbach’s α and CR values ranged from 0.972 to 0.977, exceeding the recommended threshold of 0.70. AVE values ranged from 0.896 to 0.912, all above the recommended cutoff value of 0.50.

#### 3.3.2 Foreign language enjoyment

This study employed a Foreign Language Enjoyment scale to assess learners’ enjoyment in the foreign language classroom. The scale consists of six items adapted from the original FLE scale (Dewaele and Macintyre, 2014). Sample items include “English class has a positive environment.” and “We laugh a lot in English class.” All items were rated on a five-point Likert scale ranging from “strongly disagree” to “strongly agree,” allowing for a reliable measurement of learners’ enjoyment levels. In the present study, the CFA results showed acceptable model fit: χ^2^(9) = 414.622, *p* < 0.001, GFI = 0.923, SRMR = 0.011, NFI = 0.975, TLI = 0.960, and CFI = 0.976. Although RMSEA (0.163) was above the recommended cutoff value, this may be partly due to the small degrees of freedom and the simplicity of the model. In addition, the relatively homogeneous sample may have contributed to reduced variability among indicators, which should be considered when interpreting the model fit and validity evidence.

#### 3.3.3 Academic engagement scale

This study employed the Academic Engagement Scale (Reeve and Tseng, 2011) to assess students’ engagement during learning activities, conceptualized as a multidimensional construct. The scale comprises four dimensions: behavioral engagement (5 items), agentic engagement (5 items), cognitive engagement (8 items), and emotional engagement (4 items). All items are rated on a five-point Likert scale ranging from “strongly disagree” to “strongly agree,” capturing the extent of students’ active involvement in classroom learning. For the structural model, the mean scores of each dimension were used as observed indicators of a first-order construct. In the present study, the CFA results showed generally acceptable model fit: χ^2^(203) = 3160.190, *p* < 0.001, GFI = 0.850, SRMR = 0.022, NFI = 0.953, TLI = 0.950, and CFI = 0.956. Although RMSEA (0.093) was slightly above the recommended cutoff value, the overall fit indices provided support for the adequacy of the measurement model. The constructs demonstrated excellent internal consistency and convergent validity. Cronbach’s α and CR values ranged from 0.969 to 0.983, substantially exceeding the recommended threshold of 0.70. AVE values ranged from 0.862 to 0.899, all well above the recommended criterion of 0.50, providing evidence of satisfactory convergent validity.

### 3.4 Analytical procedure

First, descriptive statistics were calculated using SPSS 28. Pearson correlation analyses were subsequently conducted in SPSS 28 to examine the relationships among the study variables. Next, structural equation modeling (SEM) was performed using the covariance-based SEM (CB-SEM) approach in SmartPLS 4.0 (Ringle et al., 2024). Although SmartPLS has traditionally been used for PLS-SEM, the current version of SmartPLS 4.0 includes a CB-SEM function. Therefore, the present study used the CB-SEM function in SmartPLS 4.0 to estimate the proposed structural model. Model fit was evaluated using several commonly reported fit indices, including χ^2^/df, CFI, TLI, RMSEA, and SRMR. Following commonly recommended criteria, χ^2^/df values below 3, CFI and TLI values of 0.90 or above, RMSEA values of.08 or below, and SRMR values of 0.05 or below were considered to indicate acceptable model fit. However, because the chi-square statistic is sensitive to sample size, model fit was interpreted based on the combined evidence from multiple fit indices rather than relying on χ^2^/df alone. The structural relationships among variables were then evaluated using standardized path coefficients, with their significance determined through bias-corrected bootstrap confidence intervals based on 5,000 resamples. Finally, mediation analysis was conducted using the CB-SEM module in SmartPLS 4.0. Indirect effects were estimated via a bias-corrected bootstrap procedure with 5,000 resamples, generating 95% confidence intervals (CIs). A mediation effect was considered statistically significant if the 95% CI did not include zero (Zhao et al., 2010). This approach does not assume normality of the sampling distribution and yields more accurate bias-corrected confidence intervals (Hayes, 2009).

## 4 Results

### 4.1 Descriptive statistics

Descriptive statistics for the main variables are presented in Table 1. Overall, students reported relatively high levels of academic engagement, with a mean score of 3.907 (SD = 0.769). Among its dimensions, behavioral engagement showed the highest mean (*M* = 3.967), followed by emotional (*M* = 3.951), cognitive (*M* = 3.920), and agentic engagement (*M* = 3.790), indicating that students were moderately to highly engaged across different aspects, though agentic engagement was comparatively lower. For GenAI feedback, the overall mean was 4.129 (SD = 0.787), suggesting generally positive perceptions. All subdimensions—accuracy (*M* = 4.147), clarity (*M* = 4.136), personalization (*M* = 4.129), and strictness (*M* = 4.106)—also showed consistently high scores, reflecting favorable evaluations of AI-generated feedback. EFL enjoyment demonstrated the highest mean among all variables (*M* = 4.283, SD = 0.737), indicating a strong positive emotional experience in the classroom. In terms of distribution, skewness and kurtosis values for all variables were within acceptable ranges (Kim, 2013), suggesting no serious deviations from normality.

**TABLE 1:** Descriptive statistics results.

Constructs		Mean		Std. deviation statistic	Variance statistic	Skewness		Kurtosis	
		Statistic	Std. error			statistic	Std. error	Statistic	Std. error
Academic engagement	Average	3.907	0.019	0.769	0.592	0.058	0.059	−0.911	0.119
	Behavioral	3.967	0.019	0.773	0.597	−0.095	0.059	−0.790	0.119
	Agentic	3.790	0.021	0.852	0.726	0.034	0.059	−0.835	0.119
	Cognitive	3.920	0.019	0.790	0.625	−0.011	0.059	−0.923	0.119
	Emotional	3.951	0.019	0.797	0.636	−0.082	0.059	−0.913	0.119
GenAI feedback	Average GenAI feedback	4.129	0.019	0.787	0.619	−0.476	0.059	−0.408	0.119
	Accuracy	4.147	0.020	0.816	0.666	−0.562	0.059	−0.347	0.119
	Strictness	4.106	0.020	0.827	0.685	−0.514	0.059	−0.365	0.119
	Clarity	4.136	0.020	0.805	0.648	−0.513	0.059	−0.419	0.119
	Personalization	4.129	0.020	0.810	0.657	−0.518	0.059	−0.366	0.119
EFL enjoyment	Average EFL enjoyment	4.283	0.018	0.737	0.543	−0.689	0.059	−0.166	0.119

### 4.2 Independent-samples *t*-tests

Independent-samples *t*-tests were conducted to examine gender differences in GenAI Feedback, Engagement, and EFL Enjoyment. Since Levene’s tests were significant for all three variables, the results based on unequal variances were reported. The results showed no significant gender difference in GenAI Feedback, *t*(1629.277) = 0.993, *p* = 0.321, Cohen’s *d* = 0.048, or EFL Enjoyment, *t*(1644.060) = 0.838, *p* = 0.402, Cohen’s *d* = 0.041. However, a significant gender difference was found in Engagement, *t*(1597.416) = 3.382, *p* < 0.001, Cohen’s *d* = 0.166, with male students reporting slightly higher engagement than female students. Overall, the effect size was small.

### 4.3 Correlation results

Pearson correlation analyses were conducted to examine the relationships among the constructs and their dimensions (see Table 2). Academic engagement was positively correlated with GenAI feedback (*r* = 0.599, *p* < 0.01) and EFL enjoyment (*r* = 0.625, *p* < 0.01), while GenAI feedback was also positively correlated with EFL enjoyment (*r* = 0.706, *p* < 0.01), providing preliminary support for the hypothesized relationships among academic engagement, GenAI feedback, and EFL enjoyment.

**TABLE 2:** Correlation results.

Constructs and dimensions	1	2	3
1 Academic engagement	–	–	–
2 GenAI feedback	0.599**		
3 EFL enjoyment	0.625**	0.706**	

**Correlation is significant at the 0.01 level (2-tailed).

### 4.4 Common method bias test

To assess common method bias, a single-factor CFA model was estimated by loading all measurement items onto one common factor. The results showed poor model fit, χ^2^/df = 194.165, RMSEA = 0.337, SRMR = 0.149, CFI = 0.620, and TLI = 0.551. These values were far from the commonly recommended criteria for acceptable model fit, suggesting that a single common factor could not adequately account for the covariance among the measurement items. Therefore, common method bias was unlikely to be a serious threat in the present study.

### 4.5 Structural equation modeling

The model fit indices for the estimated structural model are presented in Table 3. The results indicated that the model demonstrated an acceptable overall fit to the data with χ^2^ = 960.061, df = 74 and the χ^2^/df ratio 12.974. Given that the chi-square statistic is sensitive to sample size, and the present study involved a large sample (*N* = 1,701), the χ^2^/df ratio should not be used as the sole criterion for model fit evaluation, but rather interpreted in conjunction with other fit indices. The root mean square error of approximation (RMSEA) was 0.084, with a 90% confidence interval ranging from 0.079 to 0.089, indicating an acceptable level of fit. In addition, the goodness-of-fit index (GFI = 0.923) and adjusted goodness-of-fit index (AGFI = 0.890) were close to or above the recommended thresholds. The standardized root mean square residual (SRMR = 0.021) indicated a very good fit. Incremental fit indices were also satisfactory, with the normed fit index (NFI = 0.976), Tucker–Lewis index (TLI = 0.972), and comparative fit index (CFI = 0.977) all exceeding the recommended cutoff of 0.90. The parsimony goodness-of-fit index (PGFI = 0.650) further supported the model’s adequacy.

**TABLE 3:** Model fit statistics.

Index	Estimated model
χ^2^	960.061
df	74
*p*-value	< 0.001
χ^2^/df	12.974
RMSEA	0.084
RMSEA low 90% CI	0.079
RMSEA high 90% CI	0.089
GFI	0.923
AGFI	0.89
PGFI	0.65
SRMR	0.021
NFI	0.976
TLI	0.972
CFI	0.977

df, Degrees of freedom.

Overall, these results suggest that the proposed model provides a good and acceptable fit to the observed data.

### 4.6 Direct effect analysis

**FIGURE 2:**
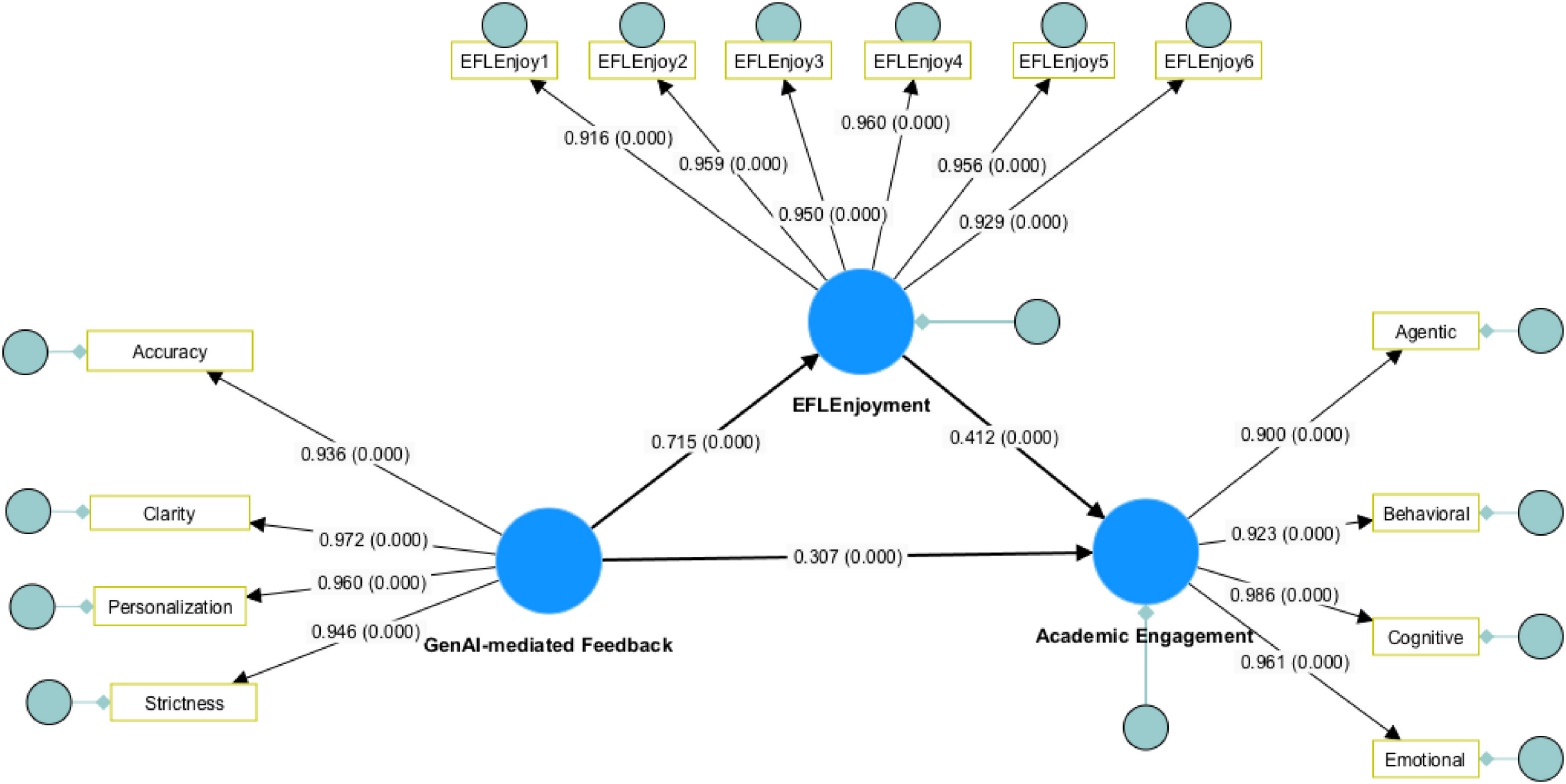
Effect tests and path analysis among various variables.

The results of the path analysis are presented in Figure 2 and Table 4. All hypothesized relationships were positive and statistically significant. Specifically, EFL enjoyment was positively associated with academic engagement [*B* = 0.442, β = 0.412, *t* = 12.453, *p* < 0.001, 95% CI (0.347, 0.474)]. Similarly, GenAI-mediated feedback was positively related to academic engagement [*B* = 0.308, β = 0.307, *t* = 8.792, *p* < 0.001, 95% CI (0.235, 0.368)]. In addition, GenAI-mediated feedback was strongly and positively associated with EFL enjoyment [B = 0.670, β = 0.715, *t* = 28.288, *p* < 0.001, 95% CI (0.671, 0.750)]. Overall, the results indicate that all variables were significantly and positively related, providing support for the hypothesized associations among GenAI-mediated feedback, EFL enjoyment, and academic engagement.

**TABLE 4:** Direct effect results.

	B	β	Standard deviation	T statistics	*P*-values	95% CI lower	95% CI upper
EFL enjoyment > academic engagement	0.442	0.412	0.035	12.453	< 0.001	0.347	0.474
GenAI-mediated feedback > academic engagement	0.308	0.307	0.035	8.792	< 0.001	0.235	0.368
GenAI-mediated feedback > EFL enjoyment	0.670	0.715	0.024	28.288	< 0.001	0.671	0.750

B, unstandardized estimate; β, standardized estimate; CI, confidence interval.

### 4.7 Mediation effect analysis

The results of the mediation analysis are presented in Table 5. The indirect relationship between GenAI-mediated feedback and academic engagement through EFL enjoyment was positive and statistically significant [*B* = 0.296, β = 0.295, *t* = 12.27, *p* < 0.001, 95% CI (0.250, 0.342)]. As the 95% confidence interval did not include zero, the indirect effect was considered statistically significant. The indirect effect accounted for approximately 49.0% of the total effect, indicating that nearly half of the association between perceptions of GenAI-mediated feedback and academic engagement was explained through foreign language enjoyment. These findings indicate that GenAI-mediated feedback was significantly associated with academic engagement via EFL enjoyment, suggesting that EFL enjoyment functioned as a significant mediating variable in the relationship between GenAI-mediated feedback and academic engagement.

**TABLE 5:** Mediation analysis results.

	B	β	Standard deviation	T statistics	*P*-values	95% CI lower	95% CI upper
GenAI-mediated Feedback > EFL Enjoyment > academic engagement	0.296	0.295	0.024	12.27	< 0.001	0.250	0.342

B, unstandardized estimate; β, standardized estimate; CI, confidence interval.

### 4.8 Total effect analysis

As shown in Table 6, the total effect of GenAI-mediated feedback on academic engagement was positive and statistically significant [*B* = 0.604, β = 0.602, *t* = 12.453, *p* < 0.001, 95% CI (0.562, 0.640)], as the 95% confidence interval did not include zero, indicating a strong overall association between the two variables. In addition, the proportion of the total effect accounted for by the indirect path through EFL enjoyment was calculated by comparing the indirect effect (β = 0.296) with the total effect (β = 0.602). The results showed that the mediating effect of EFL enjoyment accounted for approximately 49.2% of the total effect, suggesting that nearly half of the association between GenAI-mediated feedback and academic engagement was linked to EFL enjoyment.

**TABLE 6:** Total effect results.

	B	β	Standard deviation	T statistics	*P*-values	95% CI lower	95% CI upper
GenAI-mediated feedback > academic engagement	0.604	0.602	0.035	12.453	< 0.001	0.562	0.640

B, unstandardized estimate; β, standardized estimate; CI, confidence interval.

## 5 Discussion

This study aimed to examine the associations among students’ perceptions of GenAI-mediated feedback for English pronunciation practice and academic engagement, with a particular focus on the mediating role of foreign language enjoyment. Overall, the findings indicated that students’ perceptions of GenAI-mediated feedback were positively associated with both foreign language enjoyment and academic engagement. Moreover, foreign language enjoyment was also positively related to academic engagement. The results further demonstrated that foreign language enjoyment significantly mediated the relationship between GenAI-mediated feedback and academic engagement, suggesting that students who perceived GenAI feedback more favorably tended to experience higher enjoyment, which in turn was associated with greater engagement in learning activities. These findings provide empirical support for the proposed model and underscore the interconnected nature of technological feedback, emotional experience, and learner engagement in foreign language learning contexts.

### 5.1 Lower level of agentic engagement

This result suggests that, although students generally held positive perceptions of GenAI-mediated feedback and reported relatively high foreign language enjoyment, their academic engagement was comparatively more moderate. In particular, agentic engagement showed the lowest mean score among the four engagement dimensions. This pattern indicates that students’ engagement in GenAI-supported pronunciation learning may be reflected more strongly in participation, effort, emotional involvement, or cognitive investment than in agentic behaviors. In other words, students may engage with the learning tasks, but they may not necessarily take a highly proactive role in shaping, modifying, or extending the learning process.

### 5.2 Direct effect between GenAI-mediated feedback for English pronunciation practice and academic engagement

The findings of this study showed a significant positive association between students’ perceptions of GenAI-mediated feedback and academic engagement in English pronunciation practice. This result suggests that students who perceived GenAI-mediated feedback more positively tended to report higher levels of engagement in pronunciation learning activities.

The findings are highly consistent with prior research. Existing literature has long established a close and intrinsic link between feedback and student engagement, suggesting that effective feedback can enhance learners’ motivation and encourage greater investment in learning tasks (Dong et al., 2021; He and Wen, 2025; Pan and Shao, 2020).

With the advancement of educational technologies, this relationship has been further strengthened in technology-mediated feedback environments. In particular, large language model-based generative AI systems are capable of delivering immediate and personalized feedback at scale (Zhong et al., 2025). This technological innovation not only overcomes the temporal and scalability limitations of traditional human-provided feedback but also enhances students’ engagement through timely interaction, thereby fostering sustained participation and supporting continuous learning.

This relationship can be understood through the lens of Self-Determination Theory, which posits that autonomy, competence, and relatedness are three fundamental psychological needs underlying motivation and engagement in language learning. From the perspective of autonomy, AI-mediated feedback that offers meaningful choices, transparent explanations, and opportunities for self-directed adjustment can support learners’ sense of agency in speaking practice. Regarding competence, timely and targeted feedback on linguistic performance—including pronunciation, fluency, and accuracy—helps learners identify strengths and areas for improvement, thereby enhancing their perceived competence in oral communication. In terms of relatedness, interactive and responsive feedback may foster a sense of support within the learning process, partially satisfying learners’ need for social connection in language learning contexts. Together, the fulfillment of these three psychological needs is associated with increased intrinsic motivation, which in turn may promote deeper engagement in language learning.

### 5.3 Mediating role of foreign language enjoyment between GenAI-mediated feedback for English pronunciation practice and academic engagement

The findings of this study showed that foreign language enjoyment played a significant mediating role in the association between students’ perceptions of GenAI-mediated feedback and academic engagement. Specifically, students who perceived GenAI-mediated feedback more positively tended to report higher levels of foreign language enjoyment, which was in turn associated with greater academic engagement. Meanwhile, the indirect effect accounted for approximately 49.2% of the total effect, suggesting that foreign language enjoyment may represent an important emotional pathway linking students’ perceptions of GenAI-mediated feedback with academic engagement. Specifically, students who perceived GenAI-mediated feedback more positively tended to report higher levels of foreign language enjoyment, which was in turn associated with greater academic engagement. This result underscores the importance of foreign language enjoyment, indicating that fostering learners’ enjoyment may be an important way to strengthen the link between perceived GenAI-mediated feedback and academic engagement.

This finding aligns with previous research highlighting the pivotal role of positive achievement emotions in L2 learning. According to the Control Value theory (Pekrun, 2006, 2024), when learners perceive a high sense of control over the learning task (for example, through the autonomous practice and visible progress afforded by GenAI feedback) and make positive value appraisals (for example, the intrinsic satisfaction derived from improvements in pronunciation skills), they are more likely to experience higher levels of positive achievement emotions, such as foreign language enjoyment. In the present study, GenAI-mediated feedback precisely enhanced FLE by increasing learners’ perceptions of control and value.

From the perspective of the Broaden-and-Build Theory (Fredrickson, 2001, 2004), GenAI-mediated feedback serves as a primary stimulus for positive emotions. Traditional teacher-led pronunciation correction often induces high levels of anxiety and a “narrowed” focus on avoiding errors. In contrast, GenAI-mediated feedback, characterized by its immediacy, privacy, and non-judgmental nature (Zhong et al., 2025), creates a safe “playful” environment. This experience of enjoyment encourages them to try complex pronunciation or speaking tasks and engage more deeply in iterative practice. Over time, these repeated positive experiences build the learner’s self-efficacy, which further fuels their sustained engagement in oral tasks.

## 6 Implications

This study extends the applicability of the Broaden-and-Build Theory of Positive Emotions to digital learning environments. It demonstrates that AI-mediated feedback serves not merely as a cognitive tool for error correction, but also as a powerful affective driver. By stimulating positive emotions, such feedback broadens learners’ cognitive and behavioral engagement, ultimately fostering deeper and more sustained involvement in the learning process. In doing so, the study contributes to current understanding by identifying an emotional pathway linking students’ perceptions of GenAI-mediated feedback with learning-related engagement. Beyond its cognitive benefits, AI feedback emerges as a catalyst for positive emotional experiences that expand learners’ thought-action repertoires.

The findings also carry several important practical implications for the design and implementation of AI-assisted language learning systems. Pronunciation practice is often emotionally demanding because it involves repeated correction, oral production, and learners’ concerns about making errors or losing face. Therefore, AI-assisted pronunciation systems should not only function as tools for detecting and correcting pronunciation errors, but also as supportive learning environments that help learners feel safe, competent, and willing to practice repeatedly. When developing AI-based EFL platforms, designers should move beyond a narrow focus on error-correction accuracy and pay greater attention to the affective valence of the feedback provided. For example, pronunciation feedback should be delivered in a clear, encouraging, and non-judgmental manner, so that learners can interpret correction as guidance for improvement rather than as negative evaluation. Incorporating autonomy-supportive features—such as personalized goal setting and explanatory rationales—can enhance learners’ sense of agency. Furthermore, visualizing learners’ progress in meaningful and engaging ways can strengthen their perception of control and competence, thereby sustaining enjoyment, intrinsic motivation, and long-term persistence in the language learning journey. For teachers, these findings suggest that GenAI-mediated feedback may be integrated as a complementary tool in pronunciation instruction, especially for providing individualized, private, and timely feedback that is difficult to achieve in large classroom settings. In this sense, the study contributes to pronunciation research and practice by showing that the value of AI-based pronunciation feedback lies not only in technical accuracy, but also in its potential to support learners’ positive emotional experiences and engagement.

## 7 Limitations and suggestions

Despite its contributions, this study has several limitations that warrant consideration.

First, the sample was drawn from a single institution using convenience sampling through WeChat and QQ workgroups, which may limit the representativeness and generalizability of the findings. More importantly, participants shared a highly similar learning environment, grade level, curriculum, instructional materials, and exposure to GenAI-supported pronunciation learning. Such contextual homogeneity may have restricted the variability of students’ responses and amplified the empirical overlap among conceptually related dimensions, which may partly explain why the discriminant validity of some measurement instruments was not fully satisfactory in the present study. Therefore, the findings should be interpreted with caution. Future research should recruit more heterogeneous samples across different institutions, grade levels, regions, learning tasks, and instructional settings to examine whether the proposed constructs remain empirically distinguishable across broader educational contexts.

Second, the study utilized a cross-sectional design, which restricts the ability to draw causal inferences regarding the relationships among GenAI-mediated feedback, foreign language enjoyment, and academic engagement. Although significant associations were observed, the directionality of these relationships cannot be firmly established. Longitudinal or experimental designs are recommended in future studies to better investigate causal pathways and dynamic changes over time.

Third, the present study relied on students’ self-reported perceptions of GenAI-mediated feedback rather than objective records of actual feedback interactions. This was partly due to the limited accessibility of platform-based feedback data, system logs, and learners’ digital trace data, as well as privacy and ethical considerations. Therefore, the findings should be interpreted as reflecting learners’ subjective evaluations of GenAI-mediated feedback rather than the actual characteristics, frequency, or quality of the feedback itself. Future research may integrate self-report data with system-generated logs, behavioral records, or experimental intervention designs to provide a more comprehensive understanding of GenAI-mediated feedback in English pronunciation learning.

Fourth, in the present study, dimension-level mean scores of GenAI-mediated feedback and academic engagement were used as indicators to estimate the overall relationships among the main constructs. While this approach was appropriate for testing the proposed conceptual model, it did not allow us to examine whether specific feedback dimensions were differently associated with particular forms of engagement. Future research could adopt a more fine-grained analytical approach to examine the potentially differentiated relationships between feedback dimensions and engagement dimensions in GenAI-supported pronunciation learning.

Fifth, although the observed gender difference in academic engagement was relatively small, the result indicates that gender may exert a subtle but meaningful influence on learners’ engagement in language learning contexts. This suggests that individual demographic factors could shape students’ motivational and affective responses to GenAI-mediated feedback. Therefore, future studies are encouraged to incorporate gender, alongside other relevant demographic variables, as control factors when examining the relationships among GenAI-mediated feedback, foreign language enjoyment, and academic engagement, in order to obtain a more nuanced understanding of these mechanisms.

Finally, the scope of AI-mediated feedback in this study was limited to English pronunciation and speaking practice, which may constrain the generalizability of the findings to other forms of feedback in language learning. Furthermore, while the feedback scale focused specifically on pronunciation, the measures for FLE and academic engagement captured more general classroom enjoyment and learning engagement. Consequently, the observed relationships should be interpreted with caution to avoid overstating pronunciation-specific implications, as these affective and behavioral responses may reflect broader classroom dynamics rather than task-specific effects. Feedback targeting different language skills (e.g., writing, reading, or listening) may vary in its characteristics, depth, and learner perceptions, and may therefore be associated with different cognitive and affective responses. Future research could therefore examine whether the observed relationships hold across diverse feedback types and language domains using both skill-specific and generalized behavioral metrics, thereby providing a more comprehensive understanding of AI-mediated feedback in language learning.

## 8 Conclusion

The findings indicated that AI-mediated feedback is positively associated with learners’ academic engagement, both directly and indirectly through foreign language enjoyment. By integrating emotional and motivational perspectives, this study demonstrates that the effectiveness of AI feedback extends beyond cognitive support to encompass its capacity to foster positive affective experiences. These results underscore the importance of designing AI feedback systems that are not only accurate and efficient but also emotionally supportive. Overall, the study contributes to a more comprehensive understanding of how AI can facilitate language learning by simultaneously addressing learners’ cognitive and affective needs, particularly in the context of speaking development.

## Data Availability

The raw data supporting the conclusions of this article will be made available by the authors, without undue reservation.
